# Effects of alfalfa meal on quality and function of pork meatballs

**DOI:** 10.1002/fsn3.2865

**Published:** 2022-04-05

**Authors:** Zhifeng Xu, Yushuang Du, Na Li, Hongmin Geng, Qasim Ali, Xinbo Li, Yajun Gao, Yan Wang, Ronghui Xing, Jie Wu, Fangjie Cui, Chengzhang Wang, Xiaoyan Zhu, Yalei Cui, Defeng Li, Yinghua Shi

**Affiliations:** ^1^ Henan Agricultural University Zhengzhou China; ^2^ 47901 Engineering Technology Research Center for Grain & Oil Food State Administration of Grain Henan University of Technology Zhengzhou China

**Keywords:** alfalfa meal, decrease of blood lipid and body weight, quality of pork meatballs

## Abstract

Alfalfa (*Medicago sativa* L.) is abundant in dietary fiber, alfalfa saponins, and other active ingredients. However, the application of alfalfa is scarce in food. Meatball is one of the most popular meat products in daily life, but eating too many meatballs could result in obesity, hyperlipidemia, and other diseases. With increasing attention to healthy diet, how to keep the original color, aroma, taste, and shape of food with low fat and nutrition has become an urgent problem to be solved. In this study, different amounts of alfalfa meal or extruded alfalfa meal were added to pork meatballs to explore the optimal adding ratio of two kinds of alfalfa meal in pork meatballs. Further animal experiments were conducted for two weeks to prove the efficacy of two kinds of alfalfa balls in lowering blood lipid and body weight. The results showed that 0.5% alfalfa meal and 1% extruded alfalfa meal could improve the quality of prepared pork meatballs. Animal experiments demonstrated that two kinds of alfalfa meal pork meatballs had a good effect of reducing blood lipid, and the alfalfa meal pork meatballs had a better effect on reducing serum cholesterol and average daily weight gain of mice. This study provided a theoretical basis for making healthy and nutritious pork meatballs, which could provide more delicious food for people, especially people who are obese and the elderly.

## Graphical abstract

To keep the original color, aroma, taste, and shape of food with low fat and nutrition, alfalfa meal or extruded alfalfa meal was applied to pork meatballs. The research demonstrated that alfalfa meal could improve the quality of prepared pork meatballs and have a good effect on reducing blood lipid and body weight of mice.

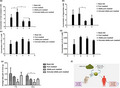

## 1 INTRODUCTION

Alfalfa (*Medicago sativa* L.) is abundant in high‐quality protein, fiber, alfalfa saponins, and mineral (such as iron, zinc, etc.; Apostol et al., 2017). Alfalfa as a dietary fiber source plays a physiological role in improving diabetes, lowering blood pressure, lowering blood lipid, and preventing cardiovascular diseases (Anderson et al., 2009; Nuttall, 1993). In daily life, dietary fiber is applied to bread, drinks, desserts, and other foods (Argiana et al., 2020; Ibrügger et al., 2012; Kurek, 2015). In addition, alfalfa saponins can also reduce the amount of blood lipid, regulate the level of cholesterol, eliminate free radicals, regulate immunity, and prevent cardiovascular diseases (Dong et al., 2007; Malinow et al., 1977, 1979). Concerning green healthy diet, researchers have begun to investigate the nutritional value and edible characteristics of alfalfa (Parrish et al., 1974).

Nowadays, meat products have become a kind of food enjoyed by millions of people, while pork meatballs are relatively common in all of them. However, the fat in common meat products reaches about 30% (Chizzolini et al.,^1999^; López‐López et al.,^2011^), which does harm to the people suffering from obesity, high blood pressure, high blood lipid, and coronary heart disease (Weinsier et al., 1976). Therefore, it has become a hot topic for nutritionists to study the way of reducing the intake of fat. Meatball, as a kind of food product that needs to be processed completely, has natural advantages in adding beneficial dietary fiber. Dietary fiber is an excellent fat substitute. For instance, when hydrated wheat was added into beef burger, the cholesterol level decreased and the wheat fiber level increased without affecting the overall palatability (Mansour & Khalil, 1997). When 5% long‐chain soluble dietary fiber is added to the sausages, it produces less heat and has better sensory ratings (Cáceres et al., 2008). When wheat bran is added to the meatballs, as the content of wheat bran increases, the water and fat content decreases (Talukder, 2015). Consequently, it is promising to combine dietary fiber with meat products. As the king of forage grass, alfalfa not only has abundant dietary fiber, but also contains many other beneficial ingredients (Apostol et al., 2017; Wang et al., 2018). Therefore, alfalfa would be the best choice for processing meatballs. How to combine alfalfa with food effectively and make it a variety of functional food needs to be further explored.

To date, there have been few studies with consistent results investigating the effects of alfalfa in pork meatballs. Therefore, in this study, a certain proportion of alfalfa meal or extruded alfalfa meal was added into pork meatballs, determining the best proportion of the two alfalfa meal in order to obtain alfalfa pork meatballs with better taste and low fat. Moreover, an animal experiment was conducted to investigate the effects of alfalfa meatball on lowering blood lipid. Thus, the alfalfa pork meatballs could become a kind of healthy functional food with low lipid. Furthermore, the addition of alfalfa meal can not only increase the economic benefits of enterprises, but also improve the health condition of people, especially those who are obese.

## 2 MATERIALS AND METHODS

### 2.1 Raw material

The formula of raw material was based on the pork's total amount of 500 g, while the ratio of fat to lean was 2:8. The extruded alfalfa meal was ground (extrusion condition: temperature 145°, extrusion moisture 22%) according to previous research with minor revision (Hernandez‐Nava et al., 2011). Both alfalfa meal and extruded alfalfa meal were passed through a 100‐mesh sieve. The addition amount of alfalfa meal or extruded alfalfa meal was variable (0.5%, 1%, 1.5%, 2%). Other raw materials were added according to the proportion in the formula (Table 1).

**TABLE 1:** The raw materials of making pork meatballs

Description	Dosage
Pork (fat/thin ratio is 2:8)	500 g
Alfalfa meal	0, 0.5%, 1%, 1.5%, 2%
Starch	75 g (15%)
Soybean protein isolate	25 g (5%)
Salt	12.5 g (2.5%)
Chicken essence	2.5 g (0.5%)
Chinese five spice	0.5 g (0.1%)
Chinese prickly ash	0.5 g (0.1%)
Black pepper powder	1 g (0.2%)
Chopped chive	10 g (2%)
Chopped ginger	5 g (1%)
Eggs	50 g (10%)
Water	150 ml (30%)
Meat elastin	2 g (0.4%)
Fresh flavor	1.5 g (0.3%)
Sesame oil	4 ml
Soy sauce	8 ml
Soybean oil	8 ml

### 2.2 Determination of the composition of alfalfa meal and extruded alfalfa meal

The ash content of alfalfa meal and extruded alfalfa meal was determined according to GB/T5505‐2008; dietary fiber was determined according to GB 5009.88‐2014; and crude protein was determined according to GB/T5511‐2008.

### 2.3 Technological process of making pork meatballs

**FIGURE 1:**
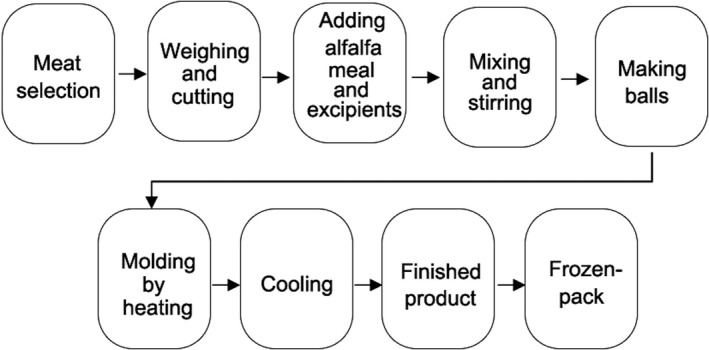
Technological flowchart of making pork meatballs

The technological process of making pork meatballs is shown in Figure 1. Select fresh boneless and peeled pork. Then pour the weighed and cut meat into the meat grinder for 1 min. Put the minced meat stuffing into a basin. First, add fresh flavor, meat elastin, salt and stir evenly in one direction, and then add a small amount of water to the meat for emulsification; add 10% eggs, alfalfa meal or extruded alfalfa meal, 15% starch, and 5% soybean protein isolate to the meat and stir in one direction; then add 0.5% chicken essence, 0.1% five‐spice powder, 0.1% prickly ash, 0.2% black pepper powder, 2% chopped chive, and 1% chopped ginger, as well as an appropriate amount of soy sauce, sesame oil, and soybean oil (Table 1). Put the mixed meat stuffing in a 4°C refrigerator for half an hour to achieve further emulsifying effects. Then squeeze it to make meatballs to keep the size uniform and the diameter of 2.5 cm. When the temperature reached about 90–95°C, the molded meatballs would be heated for 10 min. The cooked meatballs should be naturally cooled and stored in a bag.

### 2.4 Texture profile analysis of meatballs

By using the texture profile analysis (TPA) method, the characteristic parameters can be obtained by texture analyzer, such as elasticity, hardness, cohesion, and chewiness. Each formula was provided with three samples.

### 2.5 Determination of color difference of meatball samples

The values of L*, a*, and b* of sample were determined by a color difference meter. The L value is between 0 and 100, which represents the brightness coefficient. a* and b* refer to the color coefficient, which represents red‐green and yellow‐blue, respectively. If the value of a* is positive, the color is red; if the value is negative, the color is green. The larger the value is, the deeper the degree is. If b* is positive, it is yellowish, and if b* is negative, it is bluish. Δ*E* refers to the degree of difference between each value and the standard tone.

### 2.6 Sensory evaluation of meat products

Sensory evaluation is a scientific method that uses vision, taste, touch, smell, and hearing to response. It can be used to evaluate, detect, and measure the quality of products, which is the most common method to determine the taste and external quality of food. Ten evaluators were selected randomly to evaluate the color, taste, elasticity, surface texture, and taste of meatballs. In accordance with the requirements strictly, they wore the lab coat and sat in the corresponding position in the order from one to ten. Each of them was provided with a complete and half‐cut meatball, which allows the evaluator to better observe the internal compactness and roughness. The evaluation test was scored according to the criteria shown in Table 2, with a full score of 30 points.

**TABLE 2:** Sensory evaluation of pork meatballs

Description	Scoring standard	Score
Color	Uniform and bright color	5
	A little uniform color	3
	Uneven color and luster	1
Texture	Fresh, refreshing, soft, delicious, and chewable	5
	Crisp and not soft enough	3
	Soft, unshaped, and not chewable	1
Taste	A strong good taste of pork	5
	Good taste and a little taste of pork	3
	Not so good in taste	1
Tissue state	There are pork products with silk structure, solid cut surface, and uniform stomatal size	5
	The section is delicate and has no extra‐large stomata	3
	The section is not dense and the size of stomata is uneven	1
Smell	A good smell of pork	5
	A certain smell of pork	3
	Little smell of pork	1
Elasticity	It is not broken when pressed with fingers and has better elasticity and chewing ability	5
	It is not broken when pressed with fingers, but the chewing ability is general	3
	It is easily broken when pressed with fingers, and the elasticity is poor	1

### 2.7 Animal experiment

Twenty mice were divided into 4 groups with 5 mice in each group. The control group was fed basal diet. The experimental groups were fed pork meatballs, alfalfa pork meatballs, or extruded alfalfa pork meatballs for 12 h and basal diet for 12 h every day. The experiment was conducted for 2 weeks. At the end of the animal experiment, the indexes of blood lipid (total cholesterol, triglyceride, high‐ density lipoprotein cholesterol (HDL‐C), and low‐density lipoprotein cholesterol (LDL‐C)) were conducted according to manufacturer's instructions (Nanjing Jiancheng Bioengineering Institute, China). And the average daily gain of mice was calculated.

### 2.8 Statistical analysis

One‐way analysis of variance (ANOVA) was performed using SPSS 26.0 statistical software and Duncan's method was used for multiple comparisons between groups. All data were calculated as mean ± standard deviation ($\bar{x}\pm s$). A value of *p* < .05 indicates significant difference. A value of *p* < .01 indicates extremely significant difference.

## 3 RESULTS

### 3.1 Basic component of alfalfa meal

The composition of alfalfa meal used in the experiment was detected and the results are shown in Table 3. Alfalfa meal was rich in dietary fiber, with the total amount reaching 58.18%. After extrusion treatment, the proportion of soluble dietary fiber in alfalfa meal increased nearly twice.

**TABLE 3:** Component analysis of alfalfa meal before and after extrusion

Raw material	Soluble dietary fiber (%)	Insoluble dietary fiber (%)	Total dietary fiber (%)	Ash (%)	Moisture (%)	Crude protein (%)	Crude fat (%)
Alfalfa powder	2.70 ± 0.13	55.48 ± 2.26	58.18 ± 2.48	8.17 ± 0.12	5.60 ± 0.07	17.59 ± 0.65	2.41 ± 0.04
Extruded alfalfa powder	4.25 ± 0.10	43.38 ± 1.54	47.63 ± 1.49	8.04 ± 0.07	5.46 ± 0.06	20.69 ± 0.43	1.28 ± 0.05

**FIGURE 2:**
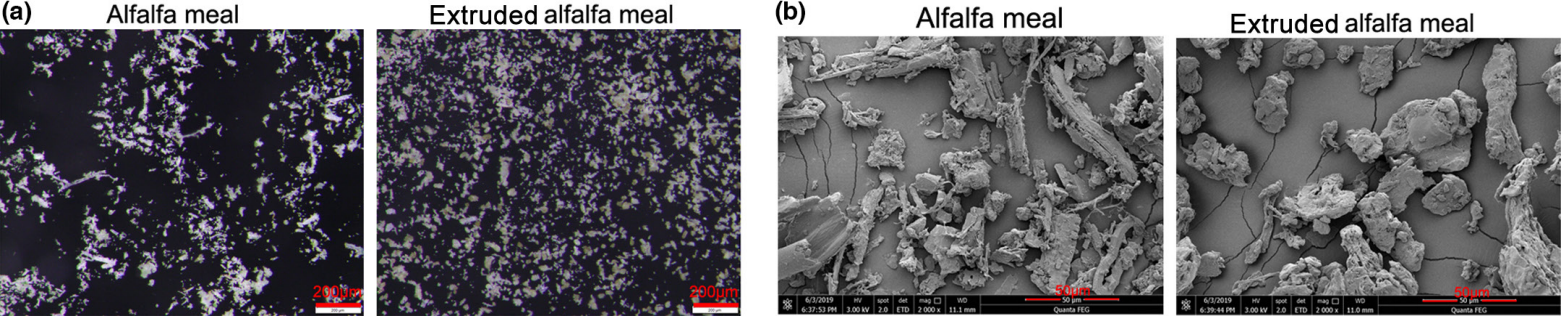
Optical photographs and scanning electron microscope (*SEM*) images of two kinds of alfalfa meal. (a) Optical photographs of alfalfa meal and extruded alfalfa meal. (b) Scanning electron microscope (*SEM*) images of alfalfa meal and extruded alfalfa meal

Figure 2a and b are optical photographs and show scanning electron microscope (*SEM*) images of alfalfa meal before and after extrusion, respectively. It can be seen from the figure that the particle size of alfalfa meal was basically less than 150 μm and there were some larger particles in extruded alfalfa meal. The color of alfalfa meal was natural green and had natural morphological structure of alfalfa, while the color of extruded alfalfa meal became dark and the morphological structure became round and similar to spherical particles.

### 3.2 Effects of alfalfa meal on the quality of pork meatballs

Alfalfa meal was added to pork meatballs according to a certain proportion, and then the color, structure, and sensory analysis of pork meatballs were tested to explore the appropriate amount of alfalfa meal in pork meatballs. The addition amount of alfalfa meal was determined to be 0.5%, 1%, 1.5%, and 2%. The texture of pork meatballs in each group was tested by the TPA. It can be seen from Table 4 that with the increasing amount of alfalfa meal, the cohesive structure of meat was damaged, resulting in a gradual increase in the hardness of meatballs, while there was a gradual decrease in elasticity and cohesion. The comprehensive effects made the chewiness of the meatball decrease gradually. But when the addition amount was 0.5%, the hardness, elasticity, and cohesive structure were acceptable.

**TABLE 4:** Effects of alfalfa meal on the texture property of pork meatballs

Amount of alfalfa meal%	0%	0.5%	1%	1.5%	2%
Hardness (g)	1599.96 ± 51.33e	2007.46 ± 96.98d	2267.76 ± 94.69c	3114.58 ± 116.44b	3330.07 ± 123.51a
Elasticity %	24.94 ± 0.51a	23.85 ± 0.482b	22.69 ± 0.32c	20.41 ± 0.53d	19.72 ± 0.61d
Cohesion	0.64 ± 0.03a	0.50 ± 0.04b	0.42 ± 0.04c	0.29 ± 0.02d	0.21 ± 0.02e
Chewiness	255.34 ± 14.59a	238.86 ± 12.16ab	216.61 ± 29.54bc	184.69 ± 20.18c	138.40 ± 20.03d

The same letters indicate no difference between groups, while different letters indicate significant difference between groups.

**FIGURE 3:**
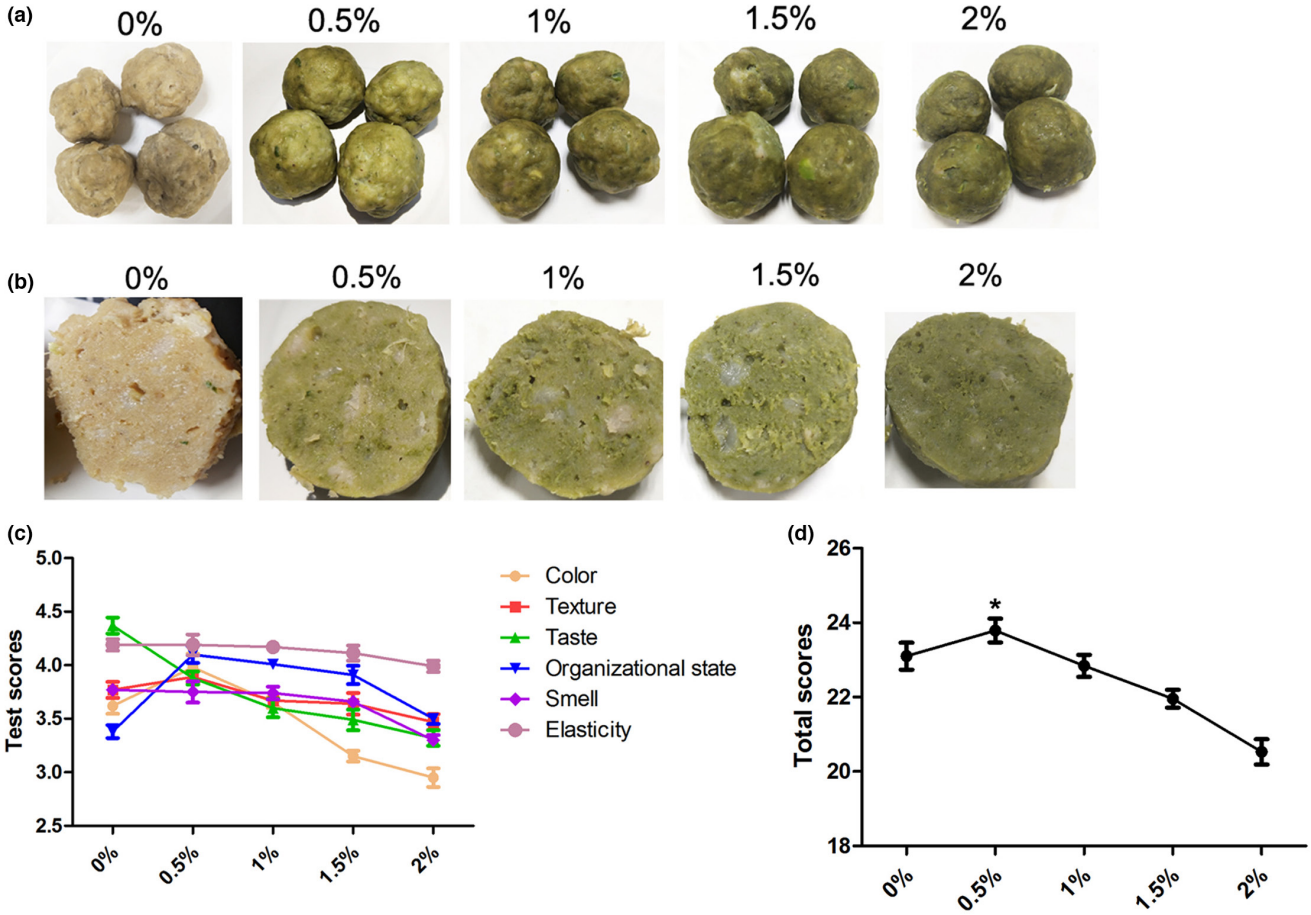
Effects of adding alfalfa meal on the quality of pork meatballs. (a) Meatballs with different amounts of alfalfa meal. (b) Section view of pork meatballs with alfalfa meal. (c) The relationship between the score of pork meatball and the adding amount of alfalfa meal

Four groups of pork meatballs were reheated and cut from the middle when they were cooled to room temperature. Then the section of the meatball was tested with a fast colorimeter. Table 5, Figure 3a, and b indicate that with the increase in the amount of alfalfa meal, the stomata of meatball were getting bigger and the color was getting darker. When the addition amount was 0.5%, the appearance was the best, the inner section had uniformly distributed tiny pores, the surface was smooth, and the color was moderate. The cut of meatballs was compact without obvious stomata when added to 0.5%. In Table 5, the brightness coefficient *L* of meatballs decreased with the increased addition of alfalfa meal, indicating that the color of meatballs was getting darker. The values of a* and b* demonstrated that the meatballs were becoming more and more yellow‐green with the increased addition of alfalfa meal.

**TABLE 5:** Effects of alfalfa meal on the color of pork meatballs

Amount of extruded alfalfa	0%	0.5%	1%	1.5%	2%
*L*	71.03 ± 0.33a	61.64 ± 0.65b	59.54 ± 0.44c	56.88 ± 0.71d	53.98 ± 0.91e
a*	5.67 ± 0.19a	2.12 ± 0.19b	−0.21 ± 0.13c	−1.65 ± 0.13d	−2.52 ± 0.20e
b*	10.80 ± 0.47d	13.71 ± 0.80c	15.10 ± 0.98bc	16.19 ± 0.66ab	17.59 ± 0.97a
Δ*E*	28.70 ± 0.95e	36.62 ± 1.06d	39.49 ± 1.14c	42.36 ± 0.92b	44.74 ± 1.10a

The same letters indicate no difference between groups, while different letters indicate significant difference between groups.

Then the sensory evaluation of each group of alfalfa meal pork meatballs was carried out, and the scores and total scores are presented in Table 6. As shown in Table 6, Figure 3c, and d, the color score of pork meatballs with 0.5% alfalfa meal was higher than that of pork meatballs without alfalfa meal. When the addition of alfalfa meal increased, the pork meatballs had a taste of crushed dregs and the tissue became rough. When added to 0.5%, the pork meatballs’ scores of color, texture, tissue state and total scores were the optimum. Therefore, it was appropriate to control the addition amount of alfalfa meal at 0.5%.

**TABLE 6:** Evaluation of the sensory property of pork meatballs by adding alfalfa meal

Amount of alfalfa meal %	0%	0.5%	1%	1.5%	2%
Color	3.62 ± 0.07b	3.98 ± 0.13a	3.65 ± 0.05b	3.15 ± 0.05c	2.95 ± 0.09d
Texture	3.77 ± 0.08ab	3.89 ± 0.05a	3.67 ± 0.05b	3.64 ± 0.10b	3.47 ± 0.07c
Taste	4.37 ± 0.08a	3.88 ± 0.06b	3.60 ± 0.09c	3.49 ± 0.10c	3.32 ± 0.07d
Tissue state	3.38 ± 0.06d	4.10 ± 0.08a	4.01 ± 0.04ab	3.91 ± 0.08b	3.50 ± 0.05c
Smell	3.77 ± 0.03a	3.75 ± 0.1a	3.74 ± 0.06a	3.66 ± 0.03a	3.30 ± 0.05b
Elasticity	4.19 ± 0.05a	4.19 ± 0.10a	4.17 ± 0.04a	4.11 ± 0.07a	3.99 ± 0.05b
Total score	23.10 ± 0.36b	23.79 ± 0.32a	22.84 ± 0.3b	21.96 ± 0.24c	20.53 ± 0.34d

The same letters indicate no difference between groups, while different letters indicate significant difference between groups.

### 3.3 Effects of extruded alfalfa meal on the quality of pork meatballs

To determine the effects of extruded alfalfa meal on the quality of pork meatballs, the addition amount of alfalfa meal was determined to be 0.5%, 1%, 1.5%, and 2%. It can be seen from Table 7 that with the increasing amount of extruded alfalfa meal addition (0.5%, 1%, 1.5%, and 2%), the hardness decreased at first and then increased. The elasticity increased at first and then decreased. Both hardness and elasticity changed when the addition amount reached up to 1%. The cohesion and chewiness in 0.5% and 1% groups had no significant difference.

**TABLE 7:** Effects of extruded alfalfa meal on the texture property of pork meatballs

Amount of extruded alfalfa meal %	0%	0.5%	1%	1.5%	2%
Hardness(g)	1599.96 ± 51.33e	2944.00 ± 142.52d	2626.79 ± 72.23c	3173.34 ± 44.5b	3377.43 ± 101.49a
Elasticity %	24.94 ± 0.51b	25.05 ± 0.38b	26.06 ± 0.69a	19.12 ± 0.27c	16.52 ± 0.35d
Cohesion	0.62 ± 0.04a	0.39 ± 0.04b	0.39 ± 0.03b	0.18 ± 0.03c	0.12 ± 0.02d
Chewiness	247.27 ± 14.29b	287.15 ± 22.09a	267.30 ± 26.28ab	109.19 ± 16.08c	67.34 ± 14.48d

The same letters indicate no difference between groups, while different letters indicate significant difference between groups.

**FIGURE 4:**
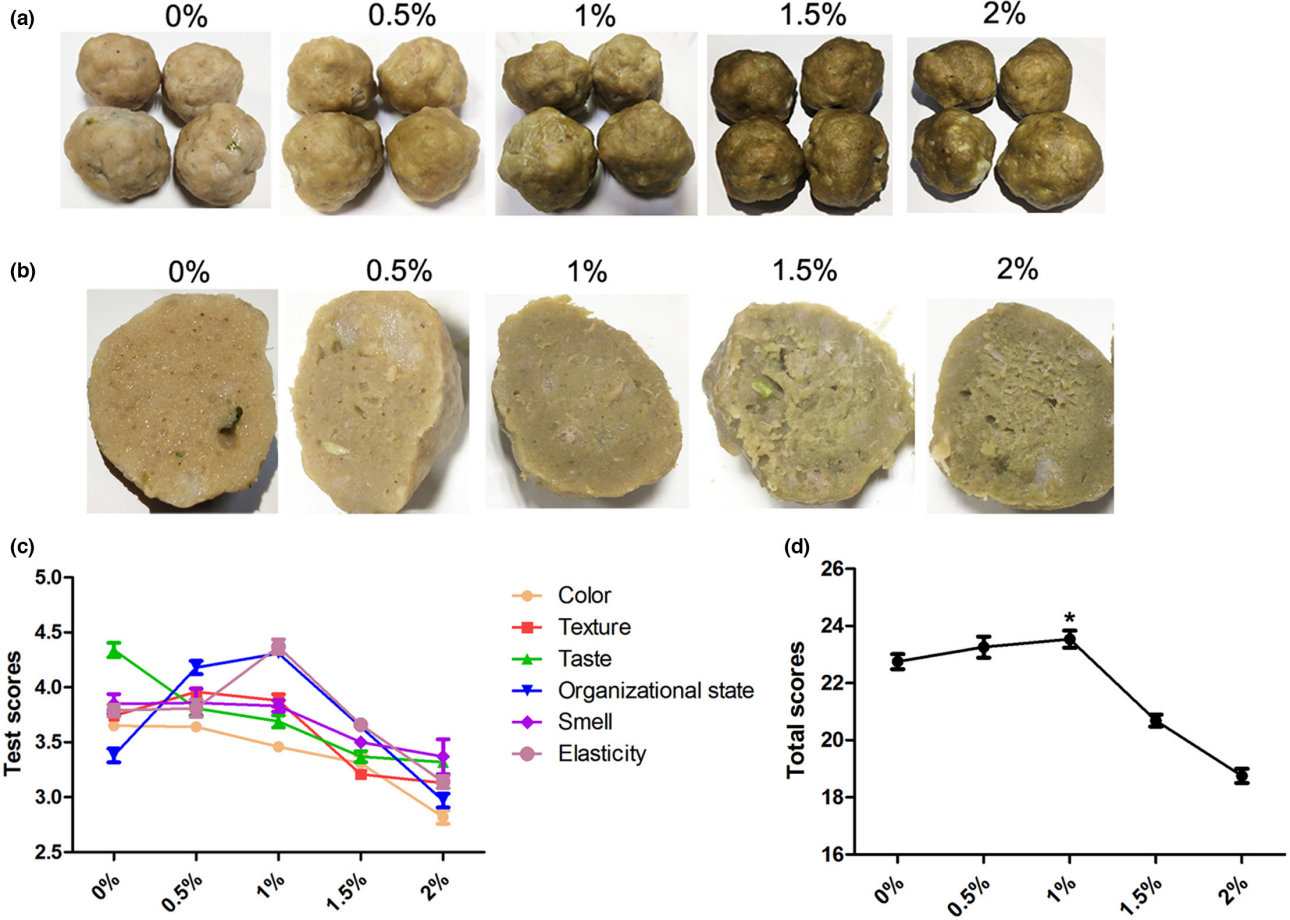
Effects of adding extruded alfalfa meal on the quality of pork meatballs. (a) Meatballs with different amounts of extruded alfalfa meal. (b) Section view of pork meatballs with extruded alfalfa meal. (c) The relationship between the score of pork meatball and the adding amount of extruded alfalfa meal

In the color difference analysis of Table 8, Figure 4a, and b, the brightness coefficient L value of the extruded alfalfa meal also showed a downward trend, and the color of the meatballs was more yellow than that of the previous group according to the value of b*. Furthermore, pork meatballs added with extruded alfalfa meal had rougher appearance and more air holes in the inner section.

**TABLE 8:** Effects of extruded alfalfa meal on the color of pork meatballs

Amount of extruded alfalfa meal	0%	0.5%	1%	1.5%	2%
L*	71.03 ± 0.33a	61.97 ± 0.50b	59.06 ± 0.60c	53.75 ± 0.45d	52.01 ± 0.41e
a*	5.67 ± 0.19a	3.25 ± 0.11b	3.18 ± 0.07bc	3.15 ± 0.07bc	2.97 ± 0.07c
b*	10.80 ± 0.47d	12.41 ± 0.26c	14.06 ± 0.16b	14.89 ± 0.29a	15.41 ± 0.17a
Δ*E*	28.70 ± 0.95d	35.99 ± 0.72c	38.79 ± 0.34b	45.61 ± 0.88a	46.12 ± 1.00a

The same letters indicate no difference between groups, while different letters indicate significant difference between groups.

The result of Table 9, Figure 4c, and d indicated that when the addition amount of extruded alfalfa meal was 1%, the score of tissue state and elasticity of meat balls was high. At the same time, the score of color, texture, taste, and smell of pork meatballs in 1% group was not too bad, so the comprehensive score was the best. When the addition of extruded alfalfa meal was less than 1%, the taste score of pork meatballs decreased slightly and was acceptable. However, when the addition of alfalfa meal was more than 1%, the total scores of pork meatballs decreased significantly. Therefore, combined with the above experimental data, the appropriate addition amount of extruded alfalfa was 1%.

**TABLE 9:** Evaluation of the sensory property of pork meatballs by adding extruded alfalfa meal

Amount of extruded alfalfa meal %	0%	0.5%	1%	1.5%	2%
Color	3.65 ± 0.05a	3.64 ± 0.03a	3.46 ± 0.04b	3.31 ± 0.07c	2.82 ± 0.06d
Texture	3.74 ± 0.03c	3.96 ± 0.03a	3.88 ± 0.06b	3.21 ± 0.03d	3.13 ± 0.03e
Taste	4.34 ± 0.07a	3.81 ± 0.06b	3.69 ± 0.06c	3.37 ± 0.05d	3.32 ± 0.04d
Tissue state	3.38 ± 0.06d	4.18 ± 0.06b	4.31 ± 0.04a	3.64 ± 0.04c	2.97 ± 0.06e
Smell	3.85 ± 0.09a	3.86 ± 0.13a	3.83 ± 0.05a	3.50 ± 0.05b	3.37 ± 0.16b
Elasticity	3.79 ± 0.05b	3.81 ± 0.08b	4.37 ± 0.07a	3.66 ± 0.04c	3.14 ± 0.06d
Total score	22.75 ± 0.26b	23.26 ± 0.37a	23.54 ± 0.30a	20.69 ± 0.21c	18.75 ± 0.15d

The same letters indicate no difference between groups, while different letters indicate significant difference between groups.

### 3.4 Effects of alfalfa and extruded alfalfa pork meatballs on blood lipid and body weight of mice

**FIGURE 5:**
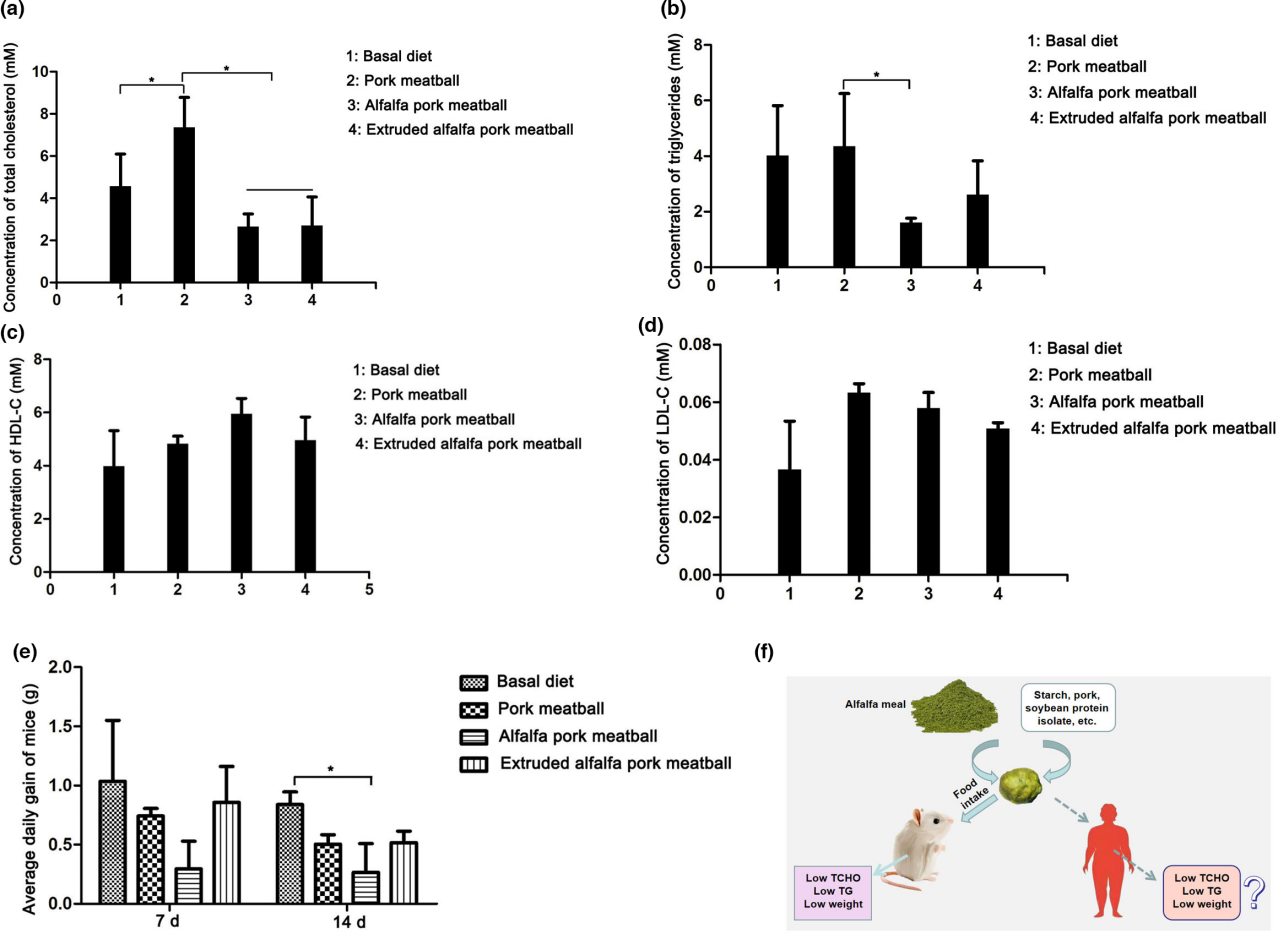
Effects of alfalfa and extruded alfalfa pork meatballs on blood lipid and weight gain of mice. (a) Effects of different treatments on the concentration of total cholesterol in serum. (b) Effects of different treatments on the concentration of triglycerides in serum. (c) Effects of different treatments on the concentration of high‐density lipoprotein cholesterol (HDL‐C) in serum. (d) Effects of different treatments on the concentration of low‐density lipoprotein cholesterol (LDL‐C) in serum. (e) Average daily weight gain of mice with different treatments. (f) Overview of the function of alfalfa pork meatballs

Mice were fed basal diet, pork meatballs, alfalfa pork meatballs, and extruded alfalfa pork meatballs. Two weeks later, the mice were sacrificed and the indexes related to blood lipid in serum were detected. Pork meatballs were shown to increase the amount of total cholesterol in serum of mice significantly (Figure 5a). But the total cholesterol concentration of mice fed 0.5% alfalfa pork meatballs and 1% extruded alfalfa pork meatball was significantly lower than that of mice fed pork meatballs (Figure 5a). The results showed that the pork meatballs added with alfalfa meal or extruded alfalfa meal played a key role in decreasing the amount of total cholesterol.

Figure 5b shows that the concentration of triglyceride in mice of alfalfa pork meatball group was significantly lower than that in the pork meatball group. The concentration of triglyceride in mice of extruded alfalfa pork meatball group was lower than that in the pork meatball group but without significance. The results indicated that the addition of alfalfa pork meatballs was better than that of extruded alfalfa pork meatballs in decreasing blood lipid, and both of them were better than pork meatballs without alfalfa meal.

As shown in Figure 5c, the concentration of HDL in serum of mice in alfalfa pork meatball group was slightly higher than that in the pork meatball group, so alfalfa pork meatball had a better effect on anti‐atherosclerosis. According to Figure 5d, the LDL concentration of mice in four treatments was not significant. Compared with the basal diet group, the average daily gain of mice at 14 days in the alfalfa pork meatball group decreased significantly, indicating that alfalfa pork meatballs can have a good effect on decreasing weight gain (Figure 5e).

In general, pork meatballs with alfalfa meal were better than pork meatballs without alfalfa meal, and alfalfa pork meatballs have a good effect on decreasing blood lipid and weight gain. The results of the present study suggested that alfalfa pork meatballs could probably improve the health condition of people who are obese through decreasing blood lipid and body weight (Figure 5f).

## 4 DISCUSSION

With improvement of life standard, there is a growing demand for healthier, more delicious, and highly nutritious food. Alfalfa is rich in fiber and essential amino acids, and contains saponins and flavonoids, as well as a high content of unsaturated fatty acids (Apostol et al., 2017; Wang et al., 2018), which is very consistent with the nutritional requirements of people. Two types of alfalfa meal, alfalfa meal and extruded alfalfa meal, were used to make pork meatballs. After extrusion, the content of total dietary fiber in alfalfa meal decreased, which may be attributed to the decomposition of some dietary fiber in the process of extrusion (Chen et al., 2018).

### 4.1 Effects of alfalfa meal on the quality of pork meatballs

Generally speaking, moderate hardness, chewiness, and elasticity of meatballs are more popular with consumers. Because the color of alfalfa is natural green of plant, the increase of its content led to the yellow‐green color of meatballs. For consumers, green meatballs can make people feel healthier, have more appetite, and would be gladly accepted by them (Tobler et al., 2011). The increasing amount of alfalfa meal resulted in higher dietary fiber that would be prone to making the compactness of meatball structure poor, loose, and fragile, resulting in poor quality. The hardness and elastic parameters of such pork meatballs were low, and the taste was not chewy and crushed. Therefore, the addition of alfalfa meal in pork meatballs should not be too high and that its addition up to 0.5% was the optimum.

### 4.2 Effects of extruded alfalfa meal on the quality of pork meatballs

With the increasing amount of extruded alfalfa meal, the hardness of meatballs decreased at first and then increased. The elasticity increased first and then decreased. Both hardness and elasticity changed when the addition amount was 1%. After extrusion, the green color of alfalfa meal was decreased, which was relatively close to the apparent color of pork meatballs. It was observed by an electron microscope that the appearance of the extruded alfalfa meal was rougher and there were more stomata in the inner section, which was due to the relatively large particles of the extruded alfalfa meal. When the addition of extruded alfalfa meal was increased to 1%, the interior section of pork meatballs contained more micropores and it was easy to be combined with pork. Therefore, the structure of pork meatballs was better when the addition of extruded alfalfa meal was 1%. The sensory property of pork meatballs decreased when the addition of extruded alfalfa meal was more than 1%, which was because of the large particles of extruded alfalfa. Therefore, the appropriate addition amount of extruded alfalfa was 1%.

### 4.3 Effects of alfalfa and extruded alfalfa pork meatballs on blood lipid and body weight of mice

Generally speaking, pork meatballs contain high fat, resulting in an increase of cholesterol and triglycerides after being eaten (Ran et al., 2020). However, the fiber in alfalfa meal has a sense of satiety and alfalfa saponins can reduce blood lipids (Clark & Slavin, 2013; Malinow et al., 1979). This may be the reason why alfalfa pork meatballs can significantly reduce the amount of total cholesterol and triglycerides in mice. Furthermore, the increase of soluble dietary fiber in extruded alfalfa pork meatballs made it easier for the body to absorb nutrients (Isken et al., 2010), which may lead to a slight reduction in the amount of triglycerides in extruded alfalfa pork meatballs. High‐ density lipoprotein cholesterol (HDL‐C), which is mainly synthesized in the liver, is an anti‐atherosclerotic lipoprotein (Zhou et al., 2015). Low‐density lipoprotein cholesterol (LDL‐C) is the main lipoprotein in fasting plasma and the main strategy for transporting cholesterol to extrahepatic tissues (Imes & Austin, 2012). Therefore, the HDL‐C increased and LDL‐C decreased in the two groups fed alfalfa pork meatballs and extruded alfalfa pork meatballs when compared with mice fed pork meatballs. Due to the efficacy of alfalfa meal, the body weight of mice in the alfalfa pork meatball group was significantly lower than that in the control group. In this study, alfalfa meal was added to pork meatballs to explore the best proportion of alfalfa meal, so as to provide a theoretical basis for the research and development of alfalfa pork meatballs in enterprises. Alfalfa pork meatballs may also reduce blood lipid level and body weight if eaten, especially by obese people, which will be in favor of their health.

## CONFLICTS OF INTEREST

The authors declare that they have no competing interests.

## Data Availability

All data is available in the manuscript.
